# Online peer support program for women with metastatic cancer: a pre-post evaluation

**DOI:** 10.1007/s00520-025-10200-w

**Published:** 2025-11-27

**Authors:** Sophia Mählmann, Hedy Kerek-Bodden, Joachim Weis

**Affiliations:** 1 Comprehensive Cancer Center Freiburg, Medical Center University of Freiburg, Hugstetter Str. 49, 79106 Freiburg, Germany; 2 Haus Der Krebs-Selbsthilfe Bundesverband, Thomas-Mann-Straße 40, 53111 Bonn, Germany

**Keywords:** Online peer support, Support groups, Women, Metastatic cancer, Evaluation, Online questionnaire

## Abstract

**Purpose:**

The diagnosis of metastatic cancer entails an enormous psychological, physical, and social burden, whereby affected women do not feel adequately supported. To address this issue, the Women’s Self-Help Organization Cancer (Frauenselbsthilfe Krebs e.V. FSH) has implemented an innovative peer support program based on online group meetings moderated by trained peers to positively influence psychosocial distress and quality of life of affected women. This pre-post non-randomized study aims to systematically evaluate this program.

**Methods:**

Participants were asked to evaluate the program in terms of expectations and concerns regarding the program, as well as experienced benefits and difficulties. We measured various psychosocial outcomes including psychosocial distress and quality of life at baseline (T0) before the program and after 6 months (T1) follow-up.

**Results:**

We included *n* = 97 patients (mostly metastatic breast cancer) at T0 and *n* = 44 patients with matching complete data sets at T1. The results showed that the program met the participants’ expectations. The online meetings were rated as helpful in terms of sharing experiences and feeling less alone. While participants initially expressed concern about confronting others’ fates (T0), this worry diminished after participation (T1). With respect to the identification of potential effects, we found a significant reduction in psychosocial stress (*t* (43) = 3.41, *p* = .001), whereas the levels of depression and anxiety remained unchanged.

**Conclusion:**

This online support program showed substantial benefits for the participants. For the next step, a controlled study should be performed to prove the effectiveness of this online program.

## Background

Women with metastatic cancer experience high levels of physical, psychological, and social burden. While the diagnosis is often associated with stigmatization, fear of progression, hopelessness, and a limited life perspective, those affected often feel inadequately supported in their situation [1–5]. In addition to professional psycho-oncological support, patient organizations can provide important peer support for affected women to cope with stressors and improve their quality of life. A peer support program enables cancer patients to share their experiences with others affected by cancer and encourage all participants to improve their knowledge and coping strategies. Moreover, it is perceived to be beneficial for the own processing of the disease and patients report greater feelings of cancer self-efficacy [6–8]. While peer support programs have proven to be helpful for many cancer patients, specific problems of women with metastatic cancer have not been sufficiently recognized or addressed in peer support programs. However, stage-specific support groups for women with breast cancer have been shown to have an advantage over mixed-stage groups [7]. In 2018/2019, a needs analysis by Weis et al. (2020) in cooperation with Women’s Self-Help Organization Cancer (Frauenselbsthilfe Krebs e.V. FSH) investigated various aspects of psychosocial distress of women with metastatic cancer. In addition, the authors explored the subjects they would like to discuss with their peers and which sort of program they would prefer [2]. The results showed that women would particularly like to exchange information and share experiences with peers on topics such as dealing with fear, death, grief, side effects, and pain. The women expressed the wish to interact in face-to-face programs (individual discussions and group meetings) or online programs (online forum/chat and online support groups). To date, only a few international studies have been conducted to investigate the effectiveness of online support groups for women with metastases. In an American study of 20 participants diagnosed with metastatic breast cancer, active participation in an online support group was shown to have similar positive effects as participation in a presence group [6]. A Cochrane systematic review of online support groups for women diagnosed with breast cancer based on six randomized controlled trials did not show superiority of online support groups compared to conventional support groups in terms of various outcome measures such as anxiety, depression, or quality of life, with the analysis showing deficiencies in sample size and quality of the studies [9]. The aim of this study is to evaluate an online peer support program with regard to various process and outcome variables. It will be examined to what extent the expectations and concerns of the participants before the start of the program are confirmed after participation. The overall effectiveness of the program is based on the assessment of the participants’ subjective benefit. Furthermore, changes in psychosocial stress, depression, anxiety symptoms, and health-related quality of life are measured before and after participation in the program.

## Methods

### Intervention

The FSH developed a new program in the form of online peer support group meetings, which allowed women with metastases to communicate via a video platform once a week. Women can participate regardless of their location, which offers great advantages for those with limited access to local support programs. During the meetings, participants can meet online, share experiences and information, and provide emotional support. If necessary, it is also possible to divide the participants into smaller subgroups in order to discuss certain topics more intensively. The meetings are moderated by women with metastatic cancer. They were recruited through a call for applications for moderators on the FSH webpage and have participated in a 2-day online moderator training conducted by a professional leadership coach and organized by the FSH. The aim of the training was to reflect on the purpose and objectives of the online meetings, to share hopes and concerns regarding moderation, and to receive methods and tools for successful moderation. It also included a technical introduction to the online tools used for the meetings. Furthermore, three psycho-oncologists accompanied the training for 1 day to facilitate an exchange about concerns as well as the handling of psychological distress during the meetings. The moderator‘s role in the online support group included empathic communication in guiding discussions, encouraging the participants to get actively involved, ensuring a respectful atmosphere, and taking organizational tasks. In order to offer participation to all interested women, the time schedules for the meetings varied over different days and day times. Once a month, the meetings were accompanied by psycho-oncologists. In addition, external health professionals were invited for online lectures addressing key topics such as pain management, sexuality, palliative care, complementary medicine, coping with anxiety, resilience, mindfulness, self-care, and communication about cancer with family and friends. Throughout the entire project period, a total of *n* = 83 online sessions were conducted.

### Study design and statistical analysis

**Fig. 1:**
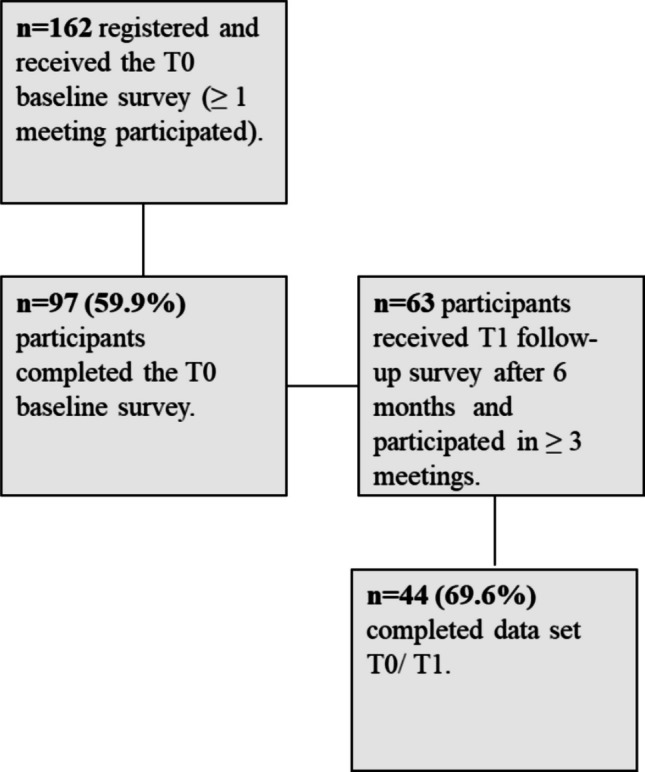
Methodological steps of data collection

The study was based on a pre-post non-randomized study design without a comparison group. Online surveys were conducted at two measurement points: prior to the individual’s first participation in a meeting (T0) and 6 months after the first meeting (T1). Data was collected between January 2021 and August 2022. This paper focusses on the evaluation of the participants. Moderators were investigated in a separate survey which will be published later. The baseline survey took place from January 2021 to March 2022. During this period, 162 participants registered for and attended at least one of the meetings. Of these, 75 women attended one or two meetings, 56 attended between three and ten meetings, and 31 attended more than ten meetings. As inclusion criteria, women who were diagnosed with metastatic cancer and were able to give consent to participate in the evaluation of the program were invited to the survey. The baseline survey was completed by 97 women. Participants were included in the T1 follow-up if they had attended at least three online meetings within the prior 6 months (*n* = 63 participants). This criterion was chosen due to the considerable variation in the structure of the meetings, which included monthly sessions with different psycho-oncologists, online lectures with different health professionals, and changing moderators in the meetings. Attending a minimum of three sessions ensured that participants had sufficient exposure to the program to provide a comprehensive evaluation. Due to missing data, we were able to achieve a complete data set for T0/T1 of *n* = 44 participants (Fig. 1).

The data was analyzed by descriptive statistical analysis. Changes in health status between the two measurement points were calculated using a *t*-test for dependent samples. The study was approved by the ethics committee of Freiburg University (approval no. 20–1314) and informed consent was obtained from all participants in the study.

### Instruments

In cooperation with the FSH, assessment parameters for the scientific evaluation were defined. The self-developed questionnaires cover questions about socio-demographics and mental health status, as well as *expectations* and *concerns* towards participation in the meetings at baseline (T0). Some items of the questionnaire at T1 match with items at T0, i.e., the extent to which the expectations were fulfilled in the form of *experiences* or the expected concerns actually occurred in the form of *difficulties*. For scaling, we used a 5-point Likert scale ranging from “strongly disagree” to “strongly agree.” Participation’s subjective benefit of the program was rated on a 5-point Likert scale from “not helpful” to “very helpful.” Furthermore, participants had the opportunity to explain their responses or add comments through open questions. In addition, standardized instruments were used to screen for psychosocial distress (NCCN Distress Thermometer [10]), depression (Patient Health Questionnaire PHQ-9 [11]), and anxiety symptoms (Generalized Anxiety Disorder Scales GAD-7 [12]) and to assess health-related quality of life (Short Form Health Survey SF-12 [13]) at both measurement points. Medical information was assessed via subjective information provided by the patients. Participants who discontinued participation in the program within the first 3 months were asked to provide reasons for dropping out via an open question.

## Results

At baseline, the average age of the women was 53.6 years (SD = 7.8). As Table 1 shows, the majority of participants (42.2%) were employed part-time or full-time. Another 37.1% (*n* = 36) of women received an early retirement or retirement pension. Only a minority of 4.1% (*n* = 4) were self-employed. The study participants were predominantly diagnosed with breast cancer (87.6%, *n* = 85), with metastases most frequent in bones (67%, *n* = 65), liver (32%, *n* = 31), and lungs (26.8%, *n* = 26). Metastases were diagnosed between May 2003 and December 2021. On average, the diagnosis of metastases dated 3.3 years back.

**Table 1:** Sociodemographic and disease-related characteristics of study participants (*n* = 97)

Characteristics at baseline (T0)	Participants (*n* = 97)	
	*n*	%
Work status		
Employee full-time	14	14.4
Employee part-time	27	27.8
Early retirement pension	26	26.8
Retirement pension	10	10.3
Self-employed	4	4.1
Other	16	16.5
Type of cancer		
Breast	85	87.6
Other*	12	12.4
Location of metastases**		
Bones	65	67.0
Liver	31	32.0
Lungs	26	26.8
Other	80	82.5

*Other types of cancer included ovarian (*n* = 4), lung (*n* = 2), intestinal (*n* = 2), pancreatic (*n* = 1), and unknown (*n* = 3) types of cancer

**Multiple responses possible; therefore, the sum of values is more than 100%

### Expectations and experiences

The results show that the participants had positive expectations regarding the program, which were mostly met after participation at T1 (Table 2). Nevertheless, there are slight differences in the degree to which the expectations were met. At T0, the study participants expressed that they would like to learn to make right decisions regarding the treatment of their disease through the online peer support meetings (M = 4.06, SD = 0.99) and to learn how to take an active role in managing their disease (M = 4.40, SD = 0.65). These expectations were only met partially in the experiences at T1 (item: decisions regarding treatment M = 3.36, SD = 0.90; active role in managing their disease M = 3.54, SD = 1.08). The expectation of feeling less alone (M = 4.27, SD = 0.87) was fully met by participation in the meetings (M = 4.47, SD = 0.72).

**Table 2:** Expectations and experiences of participants (*n* = 44)

Items I expect to… (T0), I experienced to… (T1)	T0		T1	
	M	SD	M	SD
1. … be informed about treatment options	4.20	1.04	3.40	0.97
2. … talk with other patients about cancer and related problems	4.61	0.61	4.27	0.72
3. … feel less mental distress	3.95	0.86	3.45	0.97
4. … feel less alone	4.27	0.87	4.47	0.72
5. … cope with disturbances and disagreements within the group	4.50	0.59	4.15	0.80
6. … learn to make right decisions regarding the cancer treatment	4.06	0.99	3.36	0.90
7. … learn how to take an active role in managing my disease	4.40	0.65	3.54	1.08
8. … be better prepared for consultations with physicians and therapists	3.75	1.01	3.50	0.95
9. … have more faith in the future	4.11	0.99	3.63	1.03

(1) strongly disagree, (2) disagree, (3) neither nor, (4) agree, (5) strongly agree

With regard to the overall subjective benefit of the program, 88.7% of the participants rated the meetings as “rather helpful” to “very helpful” (M = 4.45, SD = 0.81). As for the learning and experiential gains from participation, positive experiences were mentioned above all in the feeling of community and belonging, hope, confidence, courage, relief, and composure in relation to the disease. These insights emerged from the answers of the open questions. In addition, the participants rated the sessions with the psycho-oncologists as rather good to very good (M = 4.25, SD = 0.99). The sessions with presentations by external speakers were also rated positively (M = 4.50, SD = 0.62) (mean scores on a 5-point Likert scale ranging from 1 = not good at all to 5 = very good). According to the open questions, the lectures on mindfulness and self-care, an evening with relatives and loved ones, complementary medicine for alleviating side effects, laughter yoga, and palliative care were mentioned as the most popular sessions.

### Concerns and difficulties

Overall, concerns or difficulties regarding the online meetings hardly arose. Only a few concerns were indicated as relevant (see Table 3). At baseline, there was the tendency to feel burdened by confrontation with the fate of other participants (M = 3.29, SD = 1.04), which was rated slightly lower at T1 after participation in the meetings (M = 2.95, SD = 1.11). All other concerns were rather of minor importance. In particular, the concerns that participants might not feel comfortable in the group (M = 2.81, SD = 0.94), that participants might have difficulties with the handling of technology (M = 2.31, SD = 1.25), or that the protection of personal data might not be sufficient (M = 2.20, SD = 0.95) were less relevant after attending the meetings (item: not feeling comfortable in the group (M = 1.76, SD = 0.82), handling the technology (M = 1.47, SD = 0.80), or with the protection of personal data (M = 1.28, SD = 0.54)) (Table 3).

**Table 3:** Concerns and difficulties experiences by participants (*n* = 44)

Items I worry that … (T0), I experienced that… (T1)	T0		T1	
	M	SD	M	SD
1. … I do not feel comfortable in the group	2.81	0.94	1.76	0.82
2. … the fate of others could burden me	3.29	1.04	2.95	1.11
3. … there will be situations in the group that could overstrain me	2.79	1.15	2.45	1.19
4. … there will be conflicts between participants and/or moderators	2.06	0.75	1.38	0.68
5. … there will be not enough professional support	2.61	0.84	2.31	1.28
6. … the time frame of the peer support meetings will not work for me	2.56	1.06	2.11	1.10
7. … I will experience difficulties with the handling of technology (e.g. video platform, online registration)	2.31	1.25	1.47	0.80
8. … my personal data will not be sufficiently protected in the online format	2.20	0.95	1.28	0.54

(1) strongly disagree, (2) disagree, (3) neither nor, (4) agree, (5) strongly agree

The main reasons for dropping out of the meetings mentioned by the women (*n* = 41) based on the answers of the open questions were as follows: improvement or deterioration of the health condition (*n* = 11), time constraints (*n* = 7), a preference for face-to-face meetings (*n* = 5), and meetings experienced as emotionally distressing (*n* = 3). Suggestions for improvement of the online program reflected individual preferences concerning the structure of the registration and enrollment procedure, such as reminder notifications and topics of the meetings to be announced in advance. Further wishes included longer meeting durations and the repetition of sessions with specific topics presented by extermal spreakers.

### Health status

The study participants showed increased psychosocial distress on the Distress Thermometer at T0 (M = 6.70, SD = 1.87) and T1 (M = 5.52, SD = 2.39), with a threshold value for increased distress of ≥ 5 [10]. Although psychological distress remains elevated at T1, the scores have slightly decreased compared to T0. Mean scores of the screening for depressive symptoms (PHQ-9) were M = 9.18 (SD = 5.06) at T0 and M = 8.61 (SD = 4.75) at T1. Scores between 5 and 9 indicate mild depressive symptoms which means that only a small percentage was probably suffering from major depression [11]. The mean scores for the screening of anxiety symptoms (GAD-7) were lower with M = 6.88 (SD = 4.35) at T0 and M = 6.29 (SD = 4.07) at T1. Here, values from 5 to 9 also indicate mild anxiety symptoms [12]. Both mean scores are under the threshold for increased depression or anxiety levels (both ≥ 10). Regarding health-related quality of life (SF-12), the mean values on the physical sum scale (PCS) were M = 37.94 (SD = 7.15) at T0 and M = 37.16 (SD = 6.34) at T1. The mean scores of the mental sum scale (MCS) were M = 36.12 (SD = 6.74) at T0 and M = 36.77 (SD = 6.54) at T1. The physical and psychological quality of life of the participants was significantly lower on average compared to a German norm sample [14] and a mixed sample of cancer patients [15]. To estimate potential effect sizes, the comparison of the two measurement time points shows a significant decrease in mean scores only for psychosocial distress (*t* (43) = 3.41, *p* = 0.001) with a mean Cohen’s effect size [16] of *d* = 0.51. When comparing the two measurement points, there were no significant changes neither in depressive and anxiety symptoms nor in the two subscales of SF-12 for either scale (see Table 4).

**Table 4:** Health status of participants at two measurement points (*n* = 44)

Fragebögen	T0		T1				
	M	SD	M	SD	*t*(43)	*p*	Cohen’s *d*
DT^a^	6.70	1.87	5.52	2.39	3.41	**0.001**	0.51
PHQ-9^b^	9.18	5.06	8.61	4.75	0.99	0.328	0.14
GAD-7^c^	6.88	4.35	6.29	4.07	1.16	0.249	0.17
SF-12^d^							
PCS	37.94	7.15	37.16	6.34	0.80	0.427	0.12
MCS	36.12	6.74	36.77	6.54	−0.57	0.567	−0.86

^a^Distress Thermometer [10]: range 0–10

^b^*PHQ-9*, Patient Health Questionnaire [11]: sumscore-range 0–27

^c^*GAD-7*, Generalized Anxiety Disorder Scale [12]: sumscore-range 0–21

^d^*SF-12*, health-related quality of life [13] with physical sumscale (PCS) and mental sumscale (MCS)

## Discussion

Research on cancer peer support has so far only existed in its initial stages and provides partially heterogeneous results [17, 18]. This scientific evaluation of online peer support meetings for women with metastatic cancer was conducted on the basis of an explorative pre-post non-randomized study. It was shown that the program was beneficial for participants. With regard to the subjective benefit, participants reported that they profited especially from the reduction of loneliness. Further, participants gained skills in sharing experiences, which suggests for a successful fit of the program to the target group. The expressed concerns “to learn to make right decisions regarding the treatment of their disease” and “to learn how to take an active role in managing their disease” were only partially met at T1 even though participants had several opportunities to engage with medical experts through online lectures and discussions. One explanation for these findings might be that the program’s primary focus was on psychosocial and emotional support rather than disease management. Although the program offered several sessions with online lectures of medical experts, these sessions were not designed to provide individualized medical advice. Instead, they aimed to promote general understanding, self-care, and coping strategies. The screening for anxiety and depression showed that on average the women had no substantial psychiatric comorbidity, but suffered from an increased psychosocial distress level (threshold > 4). The lower values for psychosocial stress 6 months after participation in the meetings indicate that small to medium effects may have been achieved, but cannot be interpreted as evidence of the effectiveness of the program due to the explorative study design. The fact that no significant changes could be shown for anxiety, depressive symptoms, or quality of life may be an indication that the subjective benefit of this program is likely to be in the sharing of experience by the participants, whereas the fundamental problem caused by the metastatic disease could not be influenced in a relatively short follow-up period. However, these hypotheses should be tested in a controlled randomized study.

## Implications

The findings suggest that online peer support programs for women with metastatic cancer can be a feasible and well-accepted form of psychosocial support particularly to reduce feelings of loneliness and to create a sense of community and belonging. The primary benefit of the program lies in emotional and social domains rather than medical information. The partial fulfillment of expectations regarding disease management and decision-making suggests a need to better integrate medical education or to offer additional medical consultation with oncologists. The participants valued especially the sessions focusing on mindfulness, self-care, complementary medicine, laughter yoga, and palliative care, suggesting that interventions promoting well-being, emotional balance, and coping skills are of particular relevance to this target group. The popularity of the evening with relatives and loved ones also highlights the importance of including social relationships and involving family members in the supportive care concepts. Furthermore, participants expressed the wish to simplify the registration procedure, to prolong the duration of the meetings, and to repeat topic-related meetings. In line with the concept of a formative evaluation, the results were reported back to the organizers of the FSH and taken into account in the implementation of further meetings.

## Limitations

The program was designed as an online peer support program to enable flexible participation for all interested affected women. Noticeable were the irregular participation of many participants and the high dropout rate. Participants reported reasons for irregular or discontinued participation in the meetings, primarily improvement or deterioration of health condition, time constraints, a preference for face-to-face meetings, and meetings experienced as emotionally distressing. Suggestions for the improvement of the meetings referred to individual aspects of the structure of the registration and enrollment procedure, the longer duration of the meetings, and the repetition of topic-related meetings by external speakers. A high dropout rate is also observed in other studies, both in online and face-to-face peer support programs [19, 20]. We want to point out that the results of this study do not allow any conclusions about the effectiveness of the online peer support meetings as the design did not include a control group. Nevertheless, these results may provide some indications that could be helpful for further examination based on a controlled randomized study design.

## Conclusion

The newly implemented program of online peer support meetings for women with metastatic cancer was evaluated by the participants as overall helpful, especially in regard to the reduction of loneliness and the exchange of shared experiences. Against this background, the patient organization decided to prolong this online program and continued with the meetings after the completion of the evaluation. A long-term implementation of the program could offer an important addition to the professional care system to reduce the psychosocial distress of affected women. From the scientific point of view, a randomized controlled study is needed to prove the effectiveness of online peer support programs for patients with metastases.

## Data Availability

Data is available upon request, because it contains private participants informations.
